# Possible role of alpha-lipoic acid in the treatment of peripheral nerve injuries

**DOI:** 10.1186/1749-7221-5-15

**Published:** 2010-08-31

**Authors:** Maurizio Ranieri, Manuela Sciuscio, Annamaria Cortese, Marilena Stasi, Francesco Panza, Marisa Megna, Pietro Fiore, Andrea Santamato

**Affiliations:** 1 Department of Neurological and Psychiatric Sciences, Physical Medicine and Rehabilitation Unit, University of Bari "Aldo Moro", Bari, Italy; 2 Geriatric Unit & Gerontology-Geriatrics Research Laboratory, Department of Medical Sciences, IRCCS Casa Sollievo della Sofferenza, San Giovanni Rotondo, Foggia, Italy; 3 Department of Physical Medicine and Rehabilitation, University of Foggia, Foggia, Italy

## Abstract

Recent findings on the antioxidant effects of pretreatment with α-lipoic acid (α-LA) on the crush injury of rat sciatic nerve confirm the possible usefulness of α-LA administration in humans with peripheral nerve injuries. We discussed this issue in relation with our recent results in which the combined employment of α-LA and γ-linolenic acid with a rehabilitation program for six weeks reduced sensory symptoms and neuropathic pain in patients with compressive radiculopathy syndrome from disc-nerve root conflict in comparison with patients submitted to rehabilitation program alone for six weeks.

## 

Dear Editors,

We read with great interest the paper: ''Intraperitoneal alpha-lipoic acid to prevent neural damage after crush injury to the rat sciatic nerve'' by Senoglu and colleagues published in the November issue 2009 of the Journal of Brachial Plexus and Peripheral Nerve Injury [1]. We congratulate the Journal for the interest in this topic. This innovative study demonstrated the protective effect of α-lipoic acid (α-LA) administration in rat sciatic nerve crush injury by reducing the oxidative stress [1].

This paper has aroused our attention because these findings can provide confirmation on the usefulness of α-LA administration in humans in a very common disease as back pain [2], which produces adverse effects on activities of daily living. Back pain is a common and disabling musculoskeletal disorder that often occurs in a working-age population. Sciatic nerve injury is a common consequence of low back pain caused by lumbar disc herniation that occurs with radicular pain, radiculopathy, or both [3]. Nerve injury may depend on length of time of crush insult. In fact, after the tissue destruction, free oxygen radicals can increase and cause tissue damage [1]. Many patients are affected by sub-acute and chronic low back pain and/or sciatica: this exerts an important impact on the quality of life, causing interference of sleep and enjoyment of life.

Many clinical studies have compared the efficacy of surgery versus prolonged conservative treatment in the low back pain and/or sciatica [4], concluding there is no clear evidence that surgery is more beneficial than conservative treatment. Moreover often patients are affected by neuropathic pain also after the surgery. It is common knowledge that conservative treatments are tried first and surgery must be the last resort, as drug therapy (paracetamol, tramadol, nonsteroidal anti-inflammatory drugs, myorelaxant, steroids, antidepressants, etc.) and physical exercise (aerobic work, Back School, Mc Kenzie, Global Postural Re-Education, etc.) can be efficacious in the treatment of patients with back pain [4].

Recent studies showed that treatment with α-LA reduced the pain, paresthesia, and numbness in symptomatic diabetic polyneuropathy [5], and in patients with compressive radiculopathy syndrome from disc-nerve root conflict [6]. The increase of oxidative stress could be one of the causes of the nerve damage characteristic of these types of neuropathy. A sequential pattern of axonal degeneration and myelin degradation, followed by rapid regeneration is the pathophysiological mechanism into peripheral nerves after injury. It is known that excessive free radical production, if not effectively balanced by cellular antioxidant systems [superoxide dismutase (SOD) and catalase (CAT)], is responsible for aggression directed at phospholipids of membranes' cells, mitochondrial, and cellular proteins. SOD is one of protective systems against damage caused by free radicals. SOD belongs to a family of metalloproteinases and it catalyzes the dismutation of the superoxide anion radical to water and hydrogen peroxide, which is detoxified by the CAT activity [7].

α-LA is a powerful lipophilic antioxidant both in vitro and in vivo [8]. It is known to act as scavenger of many reactive oxygen species (ROS) and to interact with other antioxidants such vitamin C and vitamin E, promoting their regeneration. Therefore, α-LA has been proposed as a treatment for oxidative disorders of the nervous system characterized by an increase of free radicals.

Senoglu and colleagues, in their experimental model, obtained an important neuroprotective effect measuring the biochemical parameters (SOD, CAT activities, and malondialdehyde) on oxidative stress after nerve injury [1]. The activities of SOD and CAT were found to be high in sciatic tissue of rats indicating high production of superoxide anion radical. Therefore, the increase of SOD and CAT activities may be a response against oxidative stress. The results of Senoglu and colleagues correlated with our recent findings in which the combined employment of α-LA and γ-linolenic acid with a rehabilitation program for six weeks reduced sensory symptoms and neuropathic pain in patients with compressive radiculopathy syndrome from disc-nerve root in comparison with patients submitted to rehabilitation program alone for six weeks evaluating with Visual Analogue Scale, Short Form (36) Health Survey, Oswestry Low Back Pain Disability Questionnaire, Aberdeen Back Pain Scale, Revised Leeds Disability Questionnaire, and Roland and Morris Disability Questionnaire [6]. Although the mechanisms of nerve damage are unclear, it was hypothesized that both α-LA and SOD can increase their antioxidant protective actions. In particular, SOD could prevent the formation of ROS [7], while α-LA could act removing those just formed. Our data suggested the importance of α-LA in the treatment of peripheral nerve injuries, but further studies are needed to explain the mechanisms of its neuroprotective effects and before the use of α-LA in low back pain can be instituted, as only in diabetic neuropathy and perhaps chemotherapy-induced neuropathy has the usefulness of α-LA been proved.

## Abbreviations

**α-LA**: alpha-lipoic acid; **SOD**: superoxide dismutase; **CAT**: catalase; **ROS**: reactive oxygen species

## Competing interests

The authors declare that they have no competing interests.

## Authors' contributions

MR, FP, and AS contributed to concept, interpretation, and manuscript preparation. MS, AC, MS, MM, and PF contributed to interpretation and manuscript preparation. All authors read and approved the final manuscript.
